# Guanosine monophosphate reductase 1 is a potential therapeutic target for Alzheimer’s disease

**DOI:** 10.1038/s41598-018-21256-6

**Published:** 2018-02-09

**Authors:** Hongde Liu, Kun Luo, Donghui Luo

**Affiliations:** 1 State Key Laboratory of Bioelectronics, School of Biological Science & Medical Engineering, Southeast University, Nanjing, 210096 China; 2 Department of Neurosurgery, Xinjiang Evidence-Based Medicine Research Institute, the First Affiliated Hospital of Xinjiang Medical University, Urumqi, 830054 China; 3 Department of Neurology, the First Affiliated Hospital of Xinjiang Medical University, Urumqi, 830054 China

## Abstract

Alzheimer’s disease (AD) is a severe neurodegenerative disorder for which identification of differentially expressed genes is one way to find new therapeutic targets. Here, we conducted analysis to identify age-independent, AD-specific genes. We found that the MET, WIF1, and NPTX2 genes are downregulated in AD. WIF1 and MET are implicated in Wnt and MET signaling and regulate GSK3β activity and are thus linked with AD. Importantly, we found that the GMPR gene exhibited a gradual increase in AD progression. A logistic model based on GMPR has good ability to classify AD cases. GMPR’s product GMPR1 is in the AMPK and adenosine receptor pathways and is thus associated with Tau phosphorylation in AD. This allows GMPR1 to be a therapeutic target. Therefore, we screened five possible inhibitors to GMPR1 by docking GMPR1 with 1,174 approved drugs. Among them, lumacaftor is ideal. We then tested the effects of lumacaftor on AD model mice. After 20 days of oral administration, we observed that β-Amyloid accumulation was slowed down, and phosphorylation of Tau was almost eliminated in the treated mice. We highlight the elevated expression level of GMPR in AD and propose a therapeutic strategy of inhibiting GMPR1 with lumacaftor.

## Introduction

Alzheimer’s disease (AD), the most common cause of dementia, is characterized by extracellular amyloid plaques and intraneuronal neurofilament tangles (NFT) composed of β-amyloid protein (Aβ) and phosphorylated Tau protein, respectively^1^. AD presents a complicated pathological mechanism that is associated with multiple pathways, including the Wnt signaling, AMPK-signaling, MET signaling and A1/2 signaling pathways^2–5^, which have been implicated to play a role in Tau phosphorylation.

Glycogen synthase kinase 3 (GSK3β), one of the components of Wnt signaling, seemingly plays a central role in AD^3,6^. Activation of Wnt signaling inhibits GSK3β-mediated hyperphosphorylation of Tau protein, thus preventing the formation of NFT^3,7^. In addition, evidence has also suggested that Aβ exposure induces GSK3β activity^8^. MET signaling represses the GSK3β activity, showing crosstalk with Wnt signaling. MET contributes to nuclear translocation of β-catenin by facilitating tyrosine phosphorylation (by SRC) or by inhibition of GSK3β^9,10^. Such nuclear translocation results in transcriptional activation of Wnt ligands WNT7B and MET^3,7^, forming a feedback loop.

AMPK sensors monitor the AMP/ATP ratio (ATP level) to regulate cellular energy metabolism. It is possible that AMPK activity could decrease Aβ generation either through regulation of neuronal cholesterol and sphingomyelin levels or through upregulation of BACE1, an enzyme that cleaves amyloid precursor protein (APP)^4,11^. AMPK is also implicated in hyperphosphorylation of Tau protein^12^.

In another pathway, extracellular adenosine (A), which is generated from AMP through ecto-50-nucleotidase (CD73), binds to the A1/2 receptor, leading to an ERK-dependent increase in Tau phosphorylation and translocation towards the cytoskeleton^5,13,14^.

Identification of gene expression changes in AD will help to determine the molecular mechanisms of AD and discover new drug targets^3^. The Wnt, AMPK, MET, and A1/2 signaling pathways enrich expression-altered genes in AD, for instance, through decreased β-catenin^15^, elevated Dkk1^16^, increased A1 and A2 receptors, elevated AMP deaminase, and upregulated GSK3β^6,17^. Downregulation of NPTX2 and MET were previously reported in the literature^18^. Xiao *et al*. confirmed the reduction of NPTX2 in AD and suggested a mechanism whereby NPTX2 reduction is probably caused by increased miR-1271^19^.

The purpose of this work is to find a possible therapeutic strategy for AD based on molecular pathological mechanisms by analyzing gene expression data and screening a drug database. We focused on two issues. One was the identification of genes with different expression in AD and non-AD older adults. It is accepted that AD is a neurodegenerative disorder in older adult humans. However, AD is not found even in some older people of comparable age to patients with AD^20^. It is necessary to discriminate between age-dependent and age-independent factors in AD expression analysis, which will help to find new markers for AD. Our other focus is to find new therapeutic targets. Current therapeutic targets either enhance neurotransmitter systems or modify disease-causing pathways^2^. The latter focuses on both Aβ and NFT by modulating targets such as secretase, neutral endopeptidase, endothelin-converting enzyme, vaccination, apolipoprotein E (ApoE), GSK3β, and CDK5^21,22^.

Here, we conducted a comparative analysis to identify genes that are expressed differentially in AD. The GMPR gene, which encodes human guanosine monophosphate reductase 1 (GMPR1), was found to gradually increase its expression with AD progression. We discovered five possible inhibitors by docking GMPR1 with Food and Drug Administration (FDA)-approved drugs. We evaluated the inhibiting effect of one of the inhibitors, lumacaftor, in AD model mice. Tau phosphorylation was almost eliminated in the treated AD mice.

## Results

### Identification of age-independent differentially expressed genes

**Figure 1:**
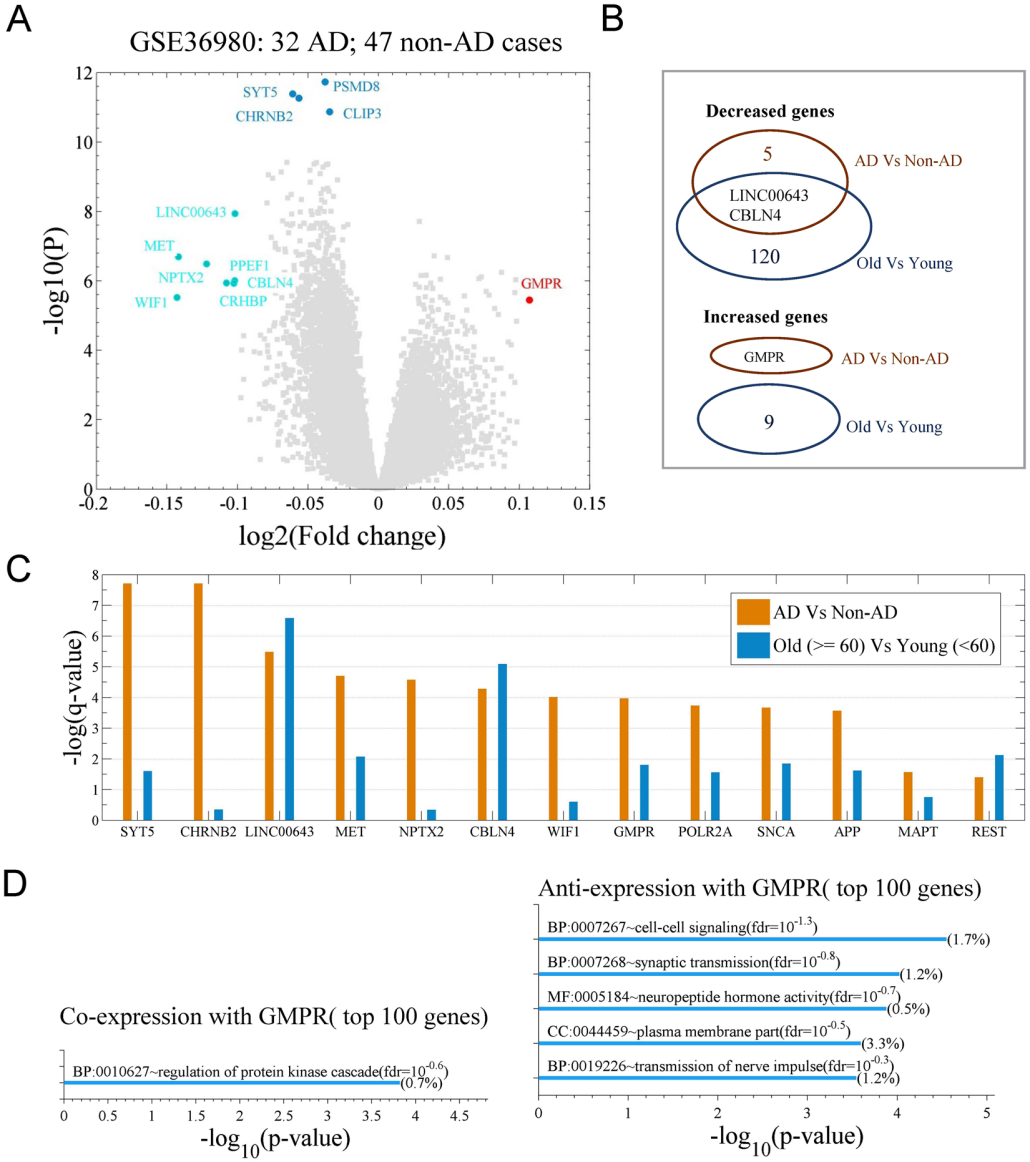
Differential expression analysis for postmortem human brain tissue of patients with Alzheimer’s disease (AD). Shown are the microarray data of 33,297 human transcripts in 32 AD samples and 47 non-AD samples (GSE36980). (**A**) Volcano plot (fold change vs. p-value (two-sample *t*-test)) for AD vs. non-AD cases; the cutoff for the differentially expressed genes is p-value ≤ 10^−5^ and log_2_ (fold change) ≥ 0.1 or ≤−0.1. (**B**) Venn plot indicates the overlapping number of genes with different expression, either in AD cases or older individuals (Figure S1A). (**C**) Q-values of genes MAPT, APP, SNCA, POLR2A, GMPR, WIF1, NPTX2, MET, LINC00643, REST, SYT5, and CHRNB2 for two comparisons (AD vs. non-AD and older vs. younger individuals). (**D**) Enrichment analysis for top 100 co-expression genes (top panel) and top 100 anti-expression genes (bottom panel) by GMPR. The analysis uses gene ontology terms provided by DAVID. The co- and anti-expression genes are identified with Pearson correlation coefficients (r) between GMPR and each gene in dataset GSE36980.

In dataset GSE36980, which includes 32 AD and 47 non-AD samples, we identified six downregulated genes and one upregulated gene according to the criteria of both p-value ≤ 10^−5^ and absolute value of log_2_ (fold change) ≥ 0.1 (Fig. 1A). In AD samples, the expression of genes NPTX2, WIF1, MET, LINC00643, CBLN4, CRHBP, and PPEF1 are downregulated. Downregulation of NPTX2 and MET were previously reported in the literatures^18,19^. Gene GMPR, which encodes protein GMPR1, is upregulated in AD cases (Fig. 1A).

SYT5, CHRNB2, PSMD8, and CLIP3 genes had significant p-values. Protein SYT5 is a calcium receptor and a negative regulator of vesicle fusion^23^. A non-coding polymorphism in CHRNB2 was reported to be associated with late-onset AD^24^. Gene PSMD8 encodes one subunit of proteasome 26S. The gene ontology of CLIP3 is related to microtubule and ganglioside binding. However, these four genes did not meet the fold change criterion and were thus not considered in the classification model.

LINC00643 and CBLN4 genes exhibit different expression between older and younger populations (GDS5204; Fig. S1A and B,C), suggesting that those genes have age-dependent expression. We noticed that the fold change in expression between AD and non-AD cases were not so great, indicating that AD probably has chronic features (Fig. S1B).

Interestingly, gene GMPR is unique because it is only an up-regulated gene in AD but not in the non-AD older population under our criterion (Fig. 1C). This suggests enzyme GMPR1, product of GMPR, is excessive in AD brain, which makes GMPR1 be a potential therapeutic target since it is easier to inhibit activity of an enzyme. Enrichment analysis indicated that GMPR’s co- and anti-expression genes have roles in cell–cell signaling, synaptic transmission, and regulation of the protein kinase cascade (Fig. 1D), implicating an important role in nerve cells.

### Classification models of AD and non-AD cases

**Figure 2:**
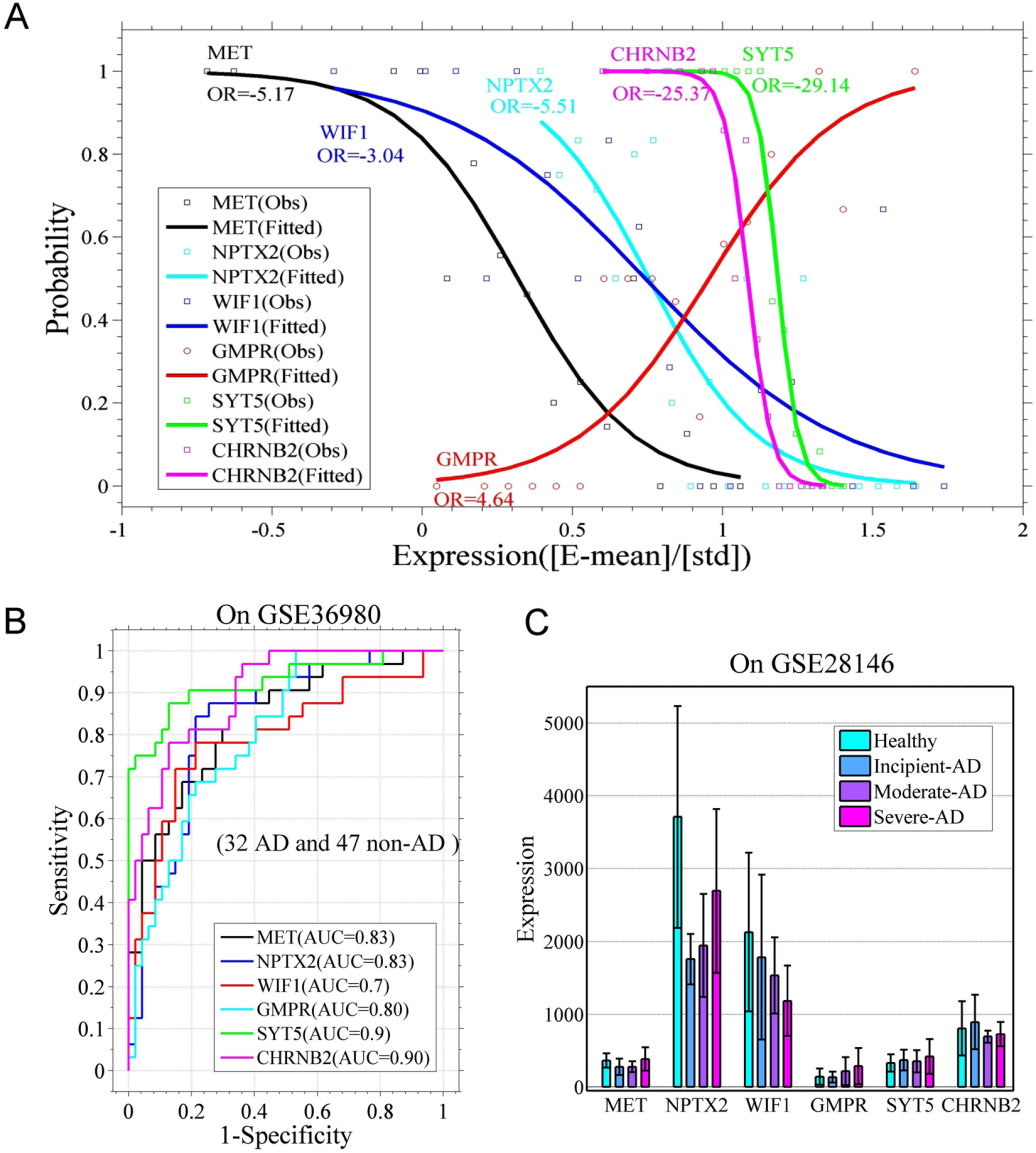
Performance of the logistic models for differentially expressed genes. (**A**) Logistic regression models for the differentially expressed genes GMPR, WIF1, NPTX2, MET, SYT5, and CHRNB2. Model parameters are listed in Tables S1 and S2. (**B**) Receiver operating characteristic (ROC) curves show models’ performance on the GSE36980 dataset (32 AD and 47 non-AD samples). AUC means area under the curve. (**C**) Average expression levels of genes GMPR, WIF1, NPTX2, MET, SYT5, and CHRNB2 in 8 Non-AD, 6 slight-AD, 9 moderate-AD, and 7 severe-AD cases (GSE28146). Error bars show standard deviations.

To evaluate the capacities of differential expression genes for AD, we constructed logistic regression models of the genes and their combinations (Fig. 2A and Tables S1–2). Models based on NPTX2, GMPR, and MET exhibited area under the curve (AUC) values of >0.8 in dataset GSE36980 (32 non-AD and 47 AD cases; Fig. 2B). The models with these gene combinations did not show obvious enhancement in terms of AUC (Fig. S2A). In dataset GSE28146 (8 non-AD and 22 AD cases), due to smaller differences between the control and incipient cases, the models showed weak classification ability (Fig. S2B).

Dataset GSE28146, in which the Mini Mental State Examination (MMSE) and NFT values were used as markers of AD progression^25,26^, is composed of 8 controls and 7 incipient, 8 moderate, and 7 severe AD cases, which allows us to observe changes in gene expression with AD progression. In this dataset, we observed a gradual increase of GMPR and a gradual decrease of WIF1 from healthy individuals to severe AD cases (Fig. 2C), and the expression alteration was highly correlated with MMSE and NFT values (Table S3, p-value ≤ 0.036), demonstrating that the two genes can be good indicators for AD progression. Importantly, the increased expression of GMPR makes the product of GMPR (GMPR1) a potential therapeutic target. Although NPTX2 expression is lower in AD cases than controls, it shows an increase from incipient to severe cases (Fig. 2C). MET, SYT5, and CHRNB2 do not show distinct expression patterns in this dataset (Fig. 2C).

Additionally, we investigated transcriptional regulation of GMPR. Among enhancers of the gene, transcription factors (TFs) JUND and CBX3 have binding motifs (Fig. S3A). In the healthy cases, GMPR and the TFs show a negative correlation (r < −0.6) in gene expression; however, in severe AD cases, they exhibit a positive correlation (r > 0.5) (Fig. S3B), indicating a reversal of the direction of transcriptional regulation in AD. Further, JUND expression is increased in AD (Fig. S3C). This means that increased GMPR expression is probably caused by abnormal transcription regulation by TF JUND.

Taken together, the results indicate that elevated GMPR levels in AD were observed in both datasets. Gene GMPR and its product GMPR1 are both potential therapeutic targets and diagnosis biomarkers.

### Links between differentially expressed genes and AD pathomechanism

**Figure 3:**
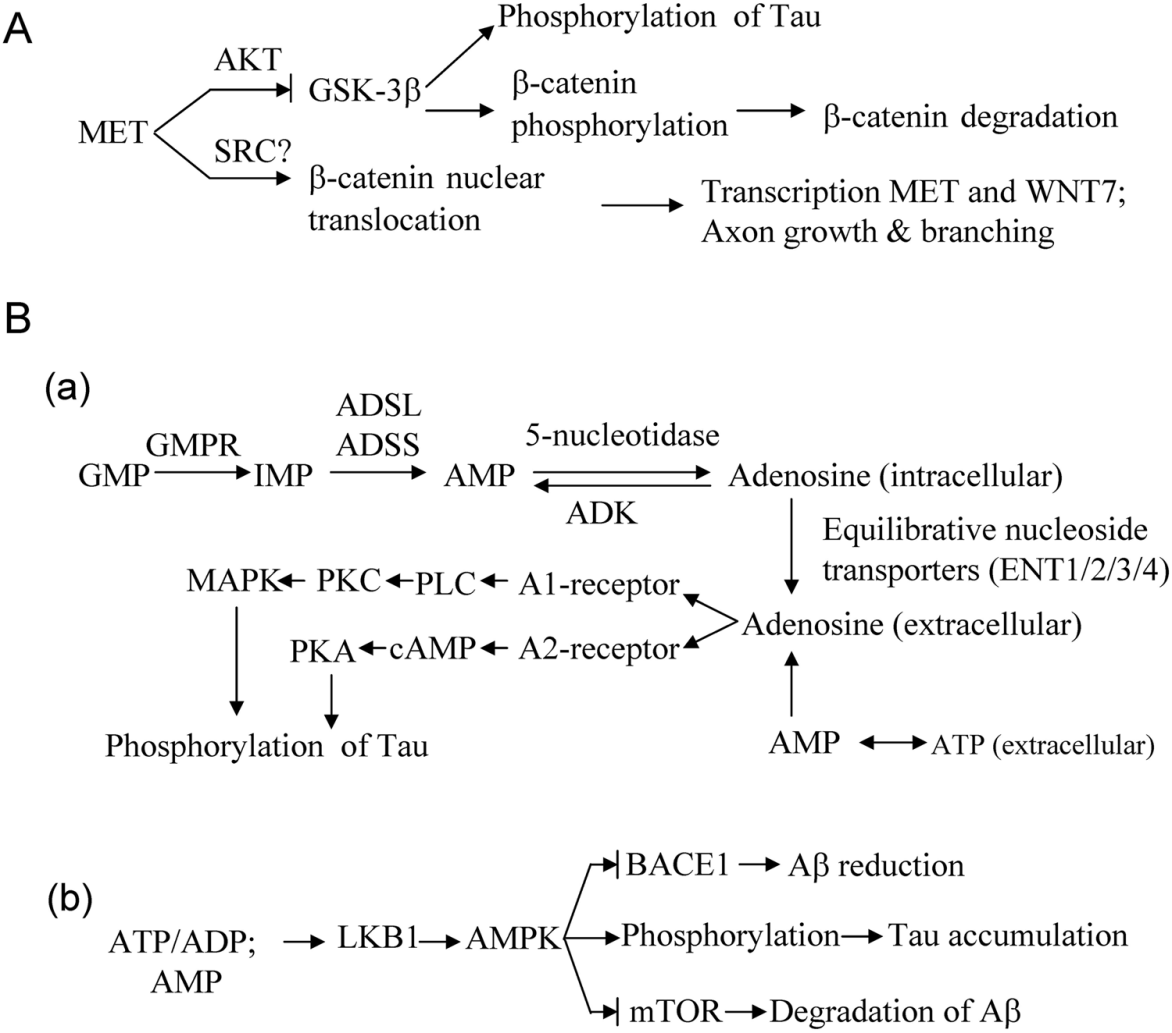
Possible mechanisms linking the differentially expressed genes and Alzheimer’s disease. (**A**) For MET. (**B**) For GMPR.

The genes with differential expression are associated with AD Aβ plaques and NFTs through multiple paths. Protein MET is a tyrosine kinase receptor that can be activated by binding of hepatocyte growth factor (HGF). MET signaling represses the GSK3β activity, which is associated with increased Tau phosphorylation^6^. MET signaling also contributes to nuclear translocation of β-catenin, consequently promoting transcription of WNT7 and MET genes (Fig. 3A)^6,9,10^. Downregulation of MET seems to facilitate AD development.

Protein GMPR1, encoded by the GMPR gene, is responsible for the conversion of GMP and NAPDH to IMP and ammonium, thus connecting purine metabolism, ATP generation, and transcriptional signaling (Fig. S4A). There exist reversible conversions between IMP and AMP and between AMP and adenosine (A)^27^. Two possible paths link GMPR and the AD phenotype. The first path is through activation of A1/A2 receptors by extracellular adenosine^27^. Extracellular adenosine results from either transmembrane transport by equilibrative nucleoside transporters or conversion of extracellular AMP (Fig. 3B). Binding of adenosine to A1/A2 receptors transduces the signaling to both PKA and PKC, thus leading to Tau phosphorylation (Fig. 3B)^14^. In a previous study, upregulation of adenosine receptors was observed in the frontal cortex in AD^14^. Further evidence has suggested that noxious brain stimuli enhance extracellular levels of adenosine^28^. It is speculated that increased GMPR levels cause adenosine accumulation, given that other enzymes cause no significant change. A dynamics simulation for the network that is constituted with the reactions shown in Fig. S4A in which we let the ATP concentration oscillate with time indicates that A levels increase with time (Fig. S4B). Further, AMP can act as a direct agonist of the A1 receptor^5^. The second possible pathway is through AMPK (Fig. 3B), which is associated with Tau accumulation and influences Aβ generation^11,12,29^. GMPR expression changes influence the levels of IMP, AMP, and ADP. It is well known that changes in the ratio of ATP to ADP or AMP can be sensed by serine/threonine-protein kinase LKB1, which then activates the AMPK pathway.

In short, upregulation of GMPR is associated with the AD phenotype through at least through two pathways: the adenosine receptor-mediated pathway and the AMPK pathway. This means the expression alteration of GMPR is probably an upstream factor that is essential to AD. Treatment targeting GMPR or its product is a possible strategy for AD.

WIF1, which inhibits Wnt signaling, is downregulated in AD, which leads to enhanced Wnt signaling, low GSK3β activity, and reduced Tau phosphorylation^7^. This means that downregulation of WIF1 is a predictive response in neuron cells.

Regarding NPTX2, the literature suggests that its reduction contributes to cognitive failure in AD through regulation of GluA4^19^. We still do not know the function of LINC00643, but it shows higher expression in the brain than in other tissue^30^.

### Inhibitors of GMPR1

**Figure 4:**
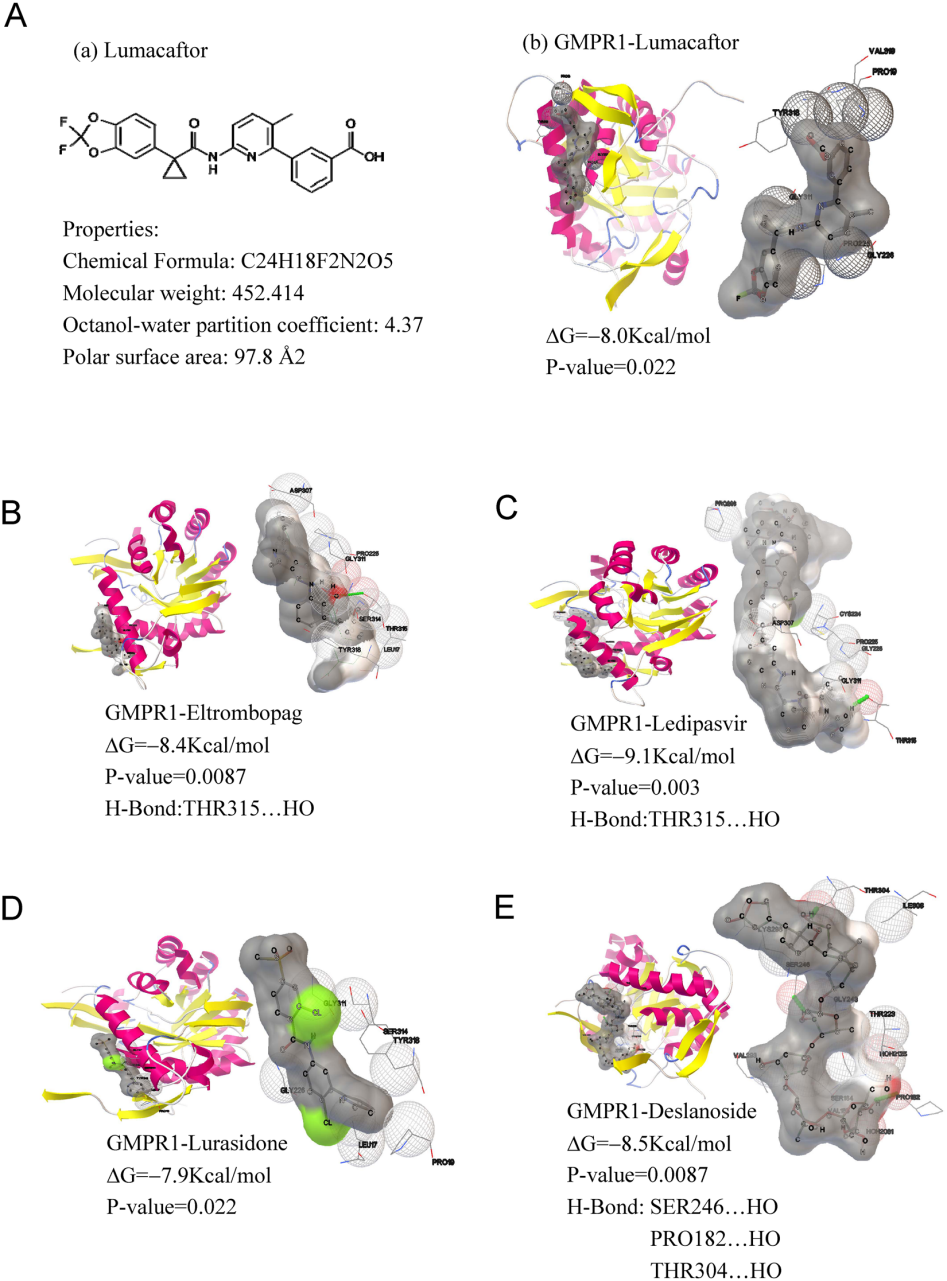
Five best potential drugs filtered by docking with human guanosine monophosphate reductase 1 (GMPR1). The crystal structure data for GMPR1 is from the Protein Data Bank (PDB ID: 2BLE, resolution: 1.9 Å). Structures of 1,174 drugs were retrieved from the DrugBank database. Docking was implemented with AutoDock Vina. GMPR1 and drug were used as receptor and ligand, respectively. The grid box accommodates all the atoms of GMPR1. The search space volume is 64 × 42 × 72 Å. The best potential drug was chosen by sorting the docking affinity values reported by AutoDock Vina. The best potential drug has the most negative free energy (ΔG). The 3D structure of each drug was generated with Babel. (**A–E**) Docked complex structures of the five ligands and GMPR1; **A**(b) Lumacaftor; (**B**) Eltrombopag; (**C**) Ledipasvir; (**D**) Lurasidone; (**E**) Deslanoside. In each panel, the left image is the full view of the complex, and the right image indicates interaction details, including the contacts between GMPR1 and the ligands. H-bonds are indicated as green sticks. ΔG means docking affinity. P-values are estimated from the distribution of docking affinities for 1,174 ligands.

To explore a therapeutic strategy, we screened for possible inhibitors of enzyme GMPR1 from 1,174 FDA-approved drugs using docking. We hoped to repurpose one of the drugs to treat AD. The 1,174 drugs, acting as ligands, were retrieved from a DrugBank^31^ dataset. Their 3D structures and atomic partial charges were computed with Babel^32^. Docking was performed by AutoDock Vina (version 1.5.6)^33^. The 1,174 drugs showed different binding affinity to GMPR1 (Fig. S5A) and were ranked by docking affinity (Fig. S5A; Table S4). By considering molecular weight and octanol-water partition coefficients (logP), five drugs were identified as candidates. Figure 4 shows the full view and interaction details for the five docked complexes. Four drugs’ interactions involve hydrogen bonds (H-bonds). Lumacaftor, which corrects the folding of cystic fibrosis transmembrane conductance regulator protein with the F508del mutation^34^, is ideal because of its small molecular weight (452), small topological polar surface area (97.8 Å^2^), and reasonable logP (4.37) (Table S4). Lumacaftor also shows strong docking with GMPR2, a paralog of GMPR1 (Fig. S5B), probably indicating that it can inhibit both GMPR1 and GMPR2. Both GMPR1 and GMPR2 function in the conversion of GMP to IMP and in the reutilization of free intracellular bases and purine nucleosides^35^. The two differ in sequence and expression levels but have a 90% identical amino acid sequence, which is also suggested by their phylogenetic tree (Fig. S6). According to the GeneCard results, GMPR1 is abundant in brain and cerebral cortex, but GMPR2 shows much lower levels (Fig. S7). Our docking results suggested that lumacaftor probably affects the GMPR2 level in other tissues.

### Therapeutic effect of lumacaftor to AD

**Figure 5:**
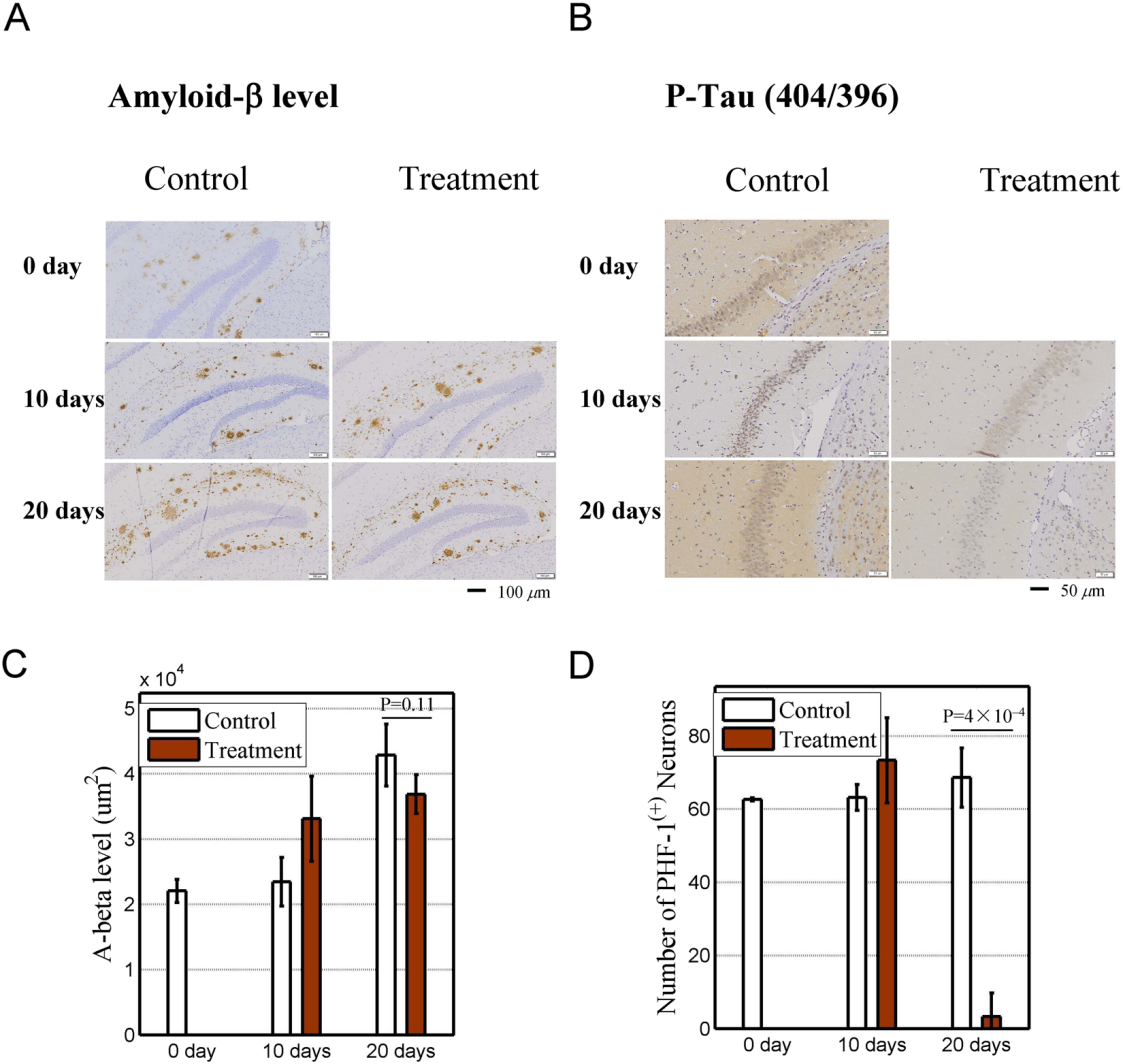
Therapeutic effect of lumacaftor on AD mice. Control group: six AD mice without lumacaftor in food. Brain tissues of groups of two mice were sectioned on the first, 10th, and 20th days after the beginning of oral drug administration, respectively. Eight total mice (treatment group) treated with lumacaftor were sectioned on the 10th and 20th days. Four mice were sacrificed each time. Levels of β-Amyloid and phosphorylated Tau are represented by averaged levels in three blindly selected zones in the parietal lobe, temporal lobe, and hippocampus. (**A**) Immunohistochemistry of β-Amyloid (brown spots) in hippocampus in control (left panels) and treatment (right panels) mice. (**B**) Same as subplot A except with phosphorylated Tau (brown + blue spots). (**C**) Average area of β-Amyloid in parietal lobe, temporal lobe, and hippocampus. Error bars indicate standard deviations. (**D**) Average number of phosphorylated Tau-positive (PHF-1^(+)^) nerve cells in parietal lobe, temporal lobe, and hippocampus. Error bars indicate standard deviations.

To this end, we tested the therapeutic effects of lumacaftor on AD model mice. The mice are 10-month-old double transgenic (APPswe & PS1dE9) model of the line B6C3-Tg (APPswePSEN1dE9Nju)/Nju^36^. Eight mice were fed with lumacaftor and six mice were used as control. The levels of Aβ and phosphorylated Tau were determined by immunohistochemistry after 0, 10, and 20 days of oral lumacaftor administration. In the hippocampus of control mice, the area of Aβ (brown spot) increased with time (left panels of Fig. 5A). In the treatment mice, the area did not increase as much as that of control mice at 20 days (right panels of Fig. 5A). Phosphorylated Tau (brown + blue spots) was drastically reduced after 20 days of treatment in mice (Fig. 5B). We estimated the average area of the Aβ spot and counted the average number of phosphorylated Tau-positive (PHF-1^(+)^) nerve cells in the parietal lobe, temporal lobe, and hippocampus (Fig. 5C,D and Tables S5–6). The results indicated that the accumulation of Aβ was greatly slowed down and that Tau phosphorylation was almost eliminated.

Taken together, these results provide evidence that GMPR1 can be a therapeutic target and that lumacaftor has therapeutic effects on AD, especially in preventing the accumulation of Aβ and eliminating Tau phosphorylation.

## Discussion

Molecular pathological mechanisms are crucial to finding effective therapeutic strategies for AD. In this work, we identified some new biomarkers and therapeutic targets by analyzing gene expression differences in AD. For one of the targets, we screened potential inhibitors and tested its therapeutic effect in AD mice. Four points should be highlighted. First, we identified the genes with different expression in AD cases than in non-AD older adults (≥60 yr). Gene expression changes represent more essential alterations in AD, as not all older people exhibit AD symptoms. Among the genes, GMPR is special because its expression increases in AD, which allows it to serve as a biomarker. The logistic model based on GMPR shows a good capacity for classification of AD cases (AUC >60% in both datasets). The model mainly shows the difference in GMPR levels between AD and non-AD cases. In practice, we cannot use cerebral tissue for diagnosis. The possible sources of specimens are cerebrospinal fluid (CSF) and peripheral blood. Thus, prior to using this model for diagnosis, it needs to be validated in CSF and peripheral blood samples. Second, the investigated genes are associated with multiple pathways. Signaling of MET and WIF1 converges on GSK3β through different pathways^3,9^. WIF1 seems to have a protective effect, as its expression is downregulated in AD, which leads to enhancement of Wnt signaling and lower phosphorylation levels^7^. GMPR1 is linked to multiple pathways, including the AMPK and adenosine (A1/A2) receptor pathways. These pathways’ activation results in phosphorylation^14,27^. An elevated GMPR1 level means a higher Tau phosphorylation level. Additionally, we inferred abnormal transcriptional regulation at one JUND-binding enhancer of GMPR in AD. All of the above indicates that GMPR1 is key node in the molecular network of AD. Third, lumacaftor (an FDA-approved drug) exhibits a high affinity and has good properties for docking with GMPR1; thus, the drug is a good candidate for a GMPR1 inhibitor. Finally, we tested the therapeutic effects of lumacaftor in AD model mice. The results showed that the drug can efficiently reduce the Tau phosphorylation in both parietal and temporal lobes and hippocampus.

## Materials and Methods

### Datasets

Three sets of expression data were used in the analysis. The first dataset, which consists of microarray expression data including postmortem human brain tissues from 32 AD cases and 47 non-AD cases, was retrieved from the literature (Gene Expression Omnibus (GEO) accession ID: GSE36980)^18^. These data were used to identify alteration of AD-specific gene expression. The second dataset included expression data from 22 older (≥60 yr, μ = 86, σ = 12) and 19 younger (<60 yr, μ = 35, σ = 9.5) cases of postmortem, neuropathologically normal, frontal cortical brain tissues. These were retrieved from the literature (GEO ID: GDS5204)^37^. This dataset was used to identify differentially expressed genes in the older population. The third dataset included 8 non-AD and 22 AD cases and was retrieved from the literature (GEO ID: GSE28146)^25^. The 22 AD cases consisted of 7 incipient cases, 8 moderate cases, and 7 severe cases. The dataset was used to validate the expression of the differential expression genes found in the first and third datasets.

The crystal structure dataset for GMPR1 was from the Protein Data Bank (PDB; accession ID: 2BLE, resolution 1.9 Å). The structures of 1,174 FDA-approved drugs were retrieved from the DrugBank database^31^. The drugs were all approved in at least one jurisdiction by 2016.

### Differential expression analysis

Two-sample *t*-tests were used to respectively detect the upregulated and downregulated genes in two datasets: GSE36980 (32 AD vs. 47 non-AD cases) and GDS5204 (22 old people vs. 19 young people). The differentially expressed genes were identified using the following criteria: p-value of *t*-test ≤10^−5^ and absolute value of log_2_ (fold change) ≥ 0.1 for dataset 1 and ≥ 0.15 for dataset 2. By comparing the two datasets in terms of differentially expressed genes, we identified age-independent differentially expressed genes in AD. A volcano plot was employed to represent both the p-values and fold changes.

Logistic regression models were constructed with the differentially expressed genes to further evaluate the differences in gene expression in datasets GSE36980 and GSE28146. Receiver operating characteristic (ROC) curves were used to show model performance.

Co-expressed genes were identified by calculating the Pearson correlation coefficient (r) of expression levels between a specific gene (e.g., GMPR) and each other gene in the first dataset. An enrichment analysis for both the top 100 co-expression genes with the GMPR gene and the top 100 anti-expression genes with GMPR was conducted on both gene ontology (GO) and KEGG pathways with the bioinformatics tool DAVID^38^.

### Screening of inhibitors for GMPR

Inhibitors of GMPR1 (PDB ID 2BLE) were screened from 1,174 FDA-approved drugs. The screening was conducted with the molecular docking tool AutoDock Vina (version 1.5.6)^33^. The atomic partial charge and 3D structure of each drug molecule were calculated with Babel (version 2.4.0) in batch mode^32^. For GMPR1’s structure, addition of hydrogen and atomic partial charge calculation were done with the AutoDockTools module (version 1.5.6). For docking, GMPR1 and the drug molecule were used as receptor and ligand, respectively. The grid box accommodated all the atoms of GMPR1, and the search space volume was 64 × 42 × 72 Å. The screening was conducted by running a script in which the AutoDock Vina executable file was repeatedly called. The best potential drug was chosen by sorting the docking affinity values reported by AutoDock Vina. The best potential drug has the most negative free energy (ΔG). Empirical p-values were estimated from the distribution of docking affinities for 1,174 ligands.

### Immunohistochemistry of β-Amyloid and phosphorylated Tau

All animal experiments followed protocols approved by the ethics committee of Nanjing Biomedical Research Institute of Nanjing University (Nanjing, China). All animal care was performed in accordance with the relevant guidelines and regulations outlined in the “Guide for Care and Use of Laboratory Animals”.

Fourteen 10-month-old B6C3-Tg (APPswePSEN1dE9Nju)/Nju mice were provided by the Nanjing Biomedical Research Institute of Nanjing University (Nanjing, China). The inhibitor lumacaftor (VX-809) was purchased from Selleck Chemicals (Texas, USA). The antibody Aβ (D12B2) Rabbit mAb #9888 was provided by CST Biological Reagents company (Shanghai, China). The antibody of Tau phosphorylated sites 404/396 (anti-PHF-1 antibody, ab184951) was purchased from Abcam (Shanghai, China).

Eight mice (i.e., the test group) were given 0.3332 mg of lumacaftor (30% PEG400 + 0.5% Tween80 + 5% Propylene glycol +64.5% double distilled water) with oil-containing food twice daily. Six mice, the control group, were fed with same food except for the addition of lumacaftor. On day 0, 10, and 20 after administration of lumacaftor, the levels of both Aβ protein and phosphorylated Tau were tested with immunohistochemistry. Aβ levels were represented by the average area positive for Aβ in three blindly selected zones in the parietal lobe, temporal lobe, and hippocampus under 10× field of view. We expected that blind selection would yield an unbiased estimation of Aβ level because we had no prior knowledge about the Aβ distribution in each mouse. Similarly, the number of neurons with phosphorylated Tau was counted in three blindly selected zones (20 × field of view) in the parietal lobe, temporal lobe, and hippocampus and then averaged.

## Electronic supplementary material

Supplementary Information
