# Supplementary material for: Guanosine monophosphate reductase 1 is a potential therapeutic target for Alzheimer’s disease
Source: Sci Rep. 2018 Feb 9;8:2759. doi: 10.1038/s41598-018-21256-6 (PMC5807363; doi:10.1038/s41598-018-21256-6)

Supplementary materials

Guanosine monophosphate reductase 1 is a potential therapeutic target for Alzheimer's disease

Hongde Liu[1*†], Kun Luo[2*†], Donghui Luo[3]

[1]State Key Laboratory of Bioelectronics, School of Biological Science & Medical Engineering, Southeast University, Nanjing 210096, China

[2]Department of Neurosurgery, Xinjiang Evidence-Based Medicine Research Institute, First Affiliated Hospital of Xinjiang Medical University, Urumqi 830054, China

[3]Department of Neurology, the First Affiliated Hospital of Xinjiang Medical University, Urumqi 830054, China

E-mails:

Hongde Liu, liuhongde@seu.edu.cn;

Kun Luo, luokun_2822@sohu.com.

Donghui Luo, dr.luodonghui@gmail.com.

* Corresponding authors. liuhongde@seu.edu.cn(HL); luokun_2822@sohu.com (KL).

† The authors contributed equally.

Tables

Table S1 Logistic regression coefficients of each single gene-based model; P-value is the hypothesis test for the coefficients.

**Gene**	**Logit Coef [β0 ;β1]**	**P–value[β0 ;β1] (*t*–test)**
MET	[1.65;–5.17]	[2×10^{-3};5×10^{-5}]
NPTX2	[4.16; –5.51]	[1×10^{-4};5×10^{-5}]
WIF1	[2.26; –3.04]	[1×10^{-3};1×10^{-5}]
GMPR	[–4.43;4.64]	[7×10^{-5};9×10^{-5}]
SYT5	[34.5; –29.14]	[2×10^{-5};1×10^{-5}]
CHRNB2	[27.49; –25.37]	[3×10^{-5};2×10^{-5}]

Table S2 Logistic regression coefficients of the models using the genes combination; P-value is the hypothesis test for the coefficients.

Genes	Logit Coef [β]	P–value [β] (*t*–test)
[GMPR; WIF1]	[6.98;12.33;–23.34]	[0.2;0.02;0.007]
[NPTX2;GMPR]	[–2.12;21.44;–16.69]	[0.8;0.02;0.06]
[MET;WIF1;SYT5]	[–82.92;7.96;18.43;104.20]	[0.001;0.4;0.02;0.01]
[MET;NPTX2;WIF1;SYT5]	[–83.31;6.77;7.60;10.49;106.13]	[0.0008;0.5;0.4;0.1;0.007]
[MET;NPTX2;GMPR;SYT5]	[–61.53;6.43;11.51;–12.87;90.90]	[0.02;0.6;0.3;0.2;0.02]
[MET;NPTX2;WIF1;GMPR;SYT5]	[–132.30;17.71;2.02;24.11;–7.25;107.19;66.47]	[0.003;0.2;0.8;0.0;0.7;0.09;0.2]

Table S3 Correlation coefficients (r) between the expression and the markers for categorizing AD progression. Listed are for GMPR and WIF1, respectively. The markers are MiniMental State Examination (MMSE) and NFT values. The data of MMSE and NFT value is from literature (Proceedings of the National Academy of Sciences of the United States of America 2004, 101(7):2173-2178.)

Genes	MMSE (r, p-value)	NFT value (r, p-value)
GMPR	[-0.99; 0.023]	[0.97, 0.024]
WIF1	[0.97; 0.022]	[-0.96, 0.036]

Table S4 Drugs with a good affinity with GMPR1; the highlighted are five ideal drugs.

Drug	Affinity (Kcal/mol)	P-value (n=1174)	Molecular formula	Molecular weight
**Ledipasvir**	**-9.10E+00**	**3.00E-03**	**C49H54F2N8O6**	**888.4134379**
Elbasvir	-8.80E+00	4.50E-03	C49H55N9O7	881.4224451
Venetoclax	-8.50E+00	8.70E-03	C45H50ClN7O7S	867.3180959
Pasireotide	-8.50E+00	8.70E-03	C58H66N10O9	1046.501424
**Deslanoside**	**-8.50E+00**	**8.70E-03**	**C47H74O19**	**942.4824302**
**Eltrombopag**	**-8.40E+00**	**8.70E-03**	**C25H22N4O4**	**442.1641052**
Suramin	-8.4	8.70E-03	C51H40N6O23S6	1296.046906
Posaconazole	-8.3	0.0129	C37H42F2N8O4	700.3297083
Ponatinib	-8.20E+00	1.29E-02	C29H27F3N6O	532.2198441
Plicamycin	-8.20E+00	1.29E-02	C52H76O24	1084.472653
Simeprevir	-8.20E+00	1.29E-02	C38H47N5O7S2	749.2916903
Lifitegrast	-8	0.0223	C29H24Cl2N2O7S	614.0681277
Ombitasvir	-8	0.0223	C50H67N7O8	893.5051121
**Lumacaftor**	**-8**	**0.0223**	**C24H18F2N2O5**	**452.118378**
Daclatasvir	-8	0.0223	C40H50N8O6	738.3853314
Regorafenib	-7.90E+00	2.23E-02	C21H15ClF4N4O3	482.0768809
**Lurasidone**	**-7.90E+00**	**2.23E-02**	**C28H36N4O2S**	**492.2558971**
Nilotinib	-7.90E+00	2.23E-02	C28H22F3N7O	529.183793

Table S5 Average area of β-Amyloid (+) in each AD mice (μm^2).

Data		Control		Treatment			
July 19, 2017	Animal ID	1943	1641	/	/	/	/
	Aβ ($^+$) Area	20759.369	23273.615	/	/	/	/
July 29, 2017	Animal ID	1950	1856	1639	75	19	52
	Aβ ($^+$) Area	26054.681	20793.338	34039.558	39110.784	35329.279	23860.400
August 8, 2017	Animal ID	1951	1931	1836	21	50	24
	Aβ ($^+$) Area	39475.639	46210.828	35653.080	33247.865	39672.366	38833.005

Table S6 Count of PHF-1$^{(+)}$ neurons in each AD mice.

Data		Control		Treatment			
July 19, 2017	Animal ID	1943	1641	/	/	/	/
	Count of PHF-1$^{(+)}$ neurons	62.89	62.33	/	/	/	/
July 29, 2017	Animal ID	1950	1856	1639	75	19	52
	Count of PHF-1$^{(+)}$ neurons	60.67	65.67	73.67	88.33	60.00	71.33
August 8, 2017	Animal ID	1951	1931	1836	21	50	24
	Count of PHF-1$^{(+)}$ neurons	74.33	62.89	0.00	0.00	0.00	13.00

Supplementary Figures

Figure legend

Figure S1 A differential expression analysis between young and old population.

A. A differential expression analysis between 22 old (≥ 60 yr, μ = 86, σ =12) and 19 young (<60 yr, μ = 35, σ =9.5) samples of the postmortem neuropathologically normal brain tissues from the frontal cortical regions (GDS5204); shown are fold change (log_2) against p-value (log10) (two-sample *t*-test). The cutoff for the differentially expressed genes is p-value ≤ 10^{-5} and either log2 (fold change) ≥ 0.15 or ≤ –0.15.

B. The fold change degree of the genes REST, MAPT, APP, SNCA, POLR2A, GMPR, WIF1, NPTX2, MET, LINC00643, SYT5 and CHRNB2 in two comparisons (AD vs non-AD and old vs. young).

Figure S2 Performance of the logistic regression models based on the combination of the genes that are different in expression in AD. The regression parameters are listed in Table S2.

A. The receiver operator characteristic (ROC) curves of the models on dataset of GSE36980 (32 AD and 47 non-AD samples). AUC means area under the curve.

B. ROCs of the models on dataset GSE28146 (8 non-AD and 22 AD samples).

Figure S3 Transcription factor JUND regulates GMPR through binding at an enhancer.

A. Shown are enhancers and transcription factors (TFs) around gene GMPR. Enhancers are indicated with black arrows. They have both acetylation of lysine 27 on histone H3 (H3K27ac) and mono- methylation of lysine 4 on histone H3 (H3K4me1).

B. Pearson correlation coefficients (r) of expression between GMPR and the gene encoding TFs. The correlation coefficients are calculated in 8 healthy cases, 7 incipient AD cases, 8 moderate AD cases and 7 severe AD cases, respectively (GSE28146). The correlation coefficient for JUND changes from a negative value in healthy cases to a positive value in severe AD cases.

C. Expression levels of genes GMPR, CBX3, JUND, ATL1, ZBTB7A, NF2L1 and CEBPB. Vertical bar indicates standard deviation error.

Figure S4 GMPR1is responsible for the reaction of GMP to IMP. Adenosine (A), AMP, ATP, ADP, GTP and other molecules connect the reaction.

A. Reactions that link with either the reactants or the resultants of the GMPR1's reaction.

B. A dynamics simulation for the network that is constituted with the reactions shown in A.

For each reaction, a partial differential equation is used to represent the relationship between reactant and resultant. Reaction rate constant is assigned in according to gene expression data of the enzymes. We let concentration of ATP oscillate with time and observe the dynamics of other molecules.

Figure S5

A, The affinity distribution of 1174 drugs in docking with GMPR1. For each drug, AutoDock Vina reports 9 conformations. The conformation with the most negative affinity was chosen and the affinity value was used to calculate the distribution.

B, The docked complex structures between human guanosine monophosphate 2 reductase 2 (GMPR2) and Lumacaftor. The crystal structure data of GMPR2 is retrieved from protein data bank (PDB ID: 2C6Q, resolution = 1.7 Å). Other computation is similar as that for GMPR1.

Figure S6 A phylogenetic tree constructed with amino acid sequences of both GMPR1 and GMPR2. The tree was constructed with bioinformatics tool MEGA4. The scale indicates evolutionary distance. Number on the leaves is from the bootstrap test (the bootstrap number is 100).

Figure S7 Shown are protein expression of both GMPR1 and GMPR2 in normal tissues and cell lines in human.

The results are retrieved from GeneCards (http://www.genecards.org/), in which the project Integrated Proteomics provides protein expression in normal tissues and cell lines from ProteomicsDB, PaxDb, MaxQB, and MOPED. (Safran M, et al, Nucl Acids Res, 2003, 31(1):142-146.)

Figure S1

A

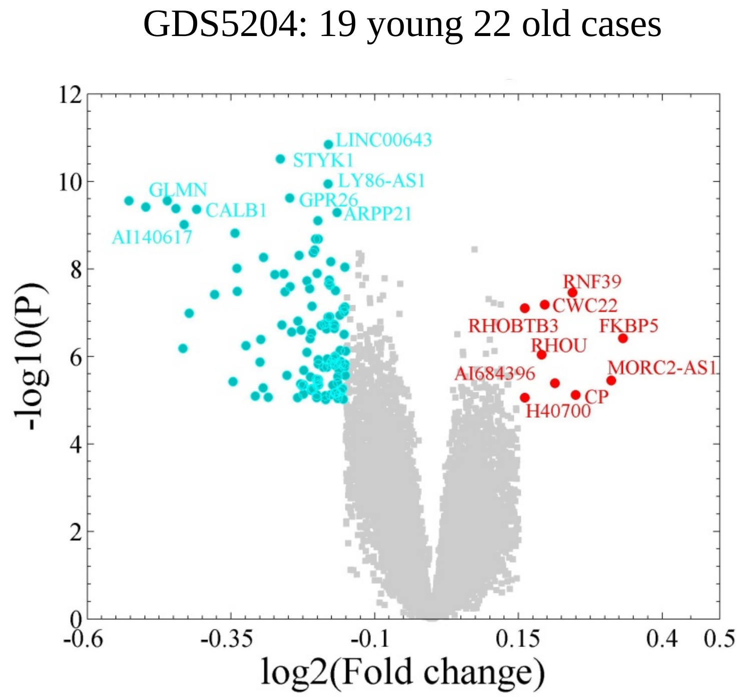
GDS5204: 19 young 22 old cases
-log10(P)
log2(Fold change)
LINC00643
STYK1
LY86-AS1
GLMN
CALB1
GPR26
ARPP21
AI140617
RNF39
CWC22
RHOBTB3
FKBP5
RHOU
AI684396
MORC2-AS1
CP
H40700
12
10
8
6
4
2
0
-0.6
-0.35
-0.1
0.15
0.4
0.5

B

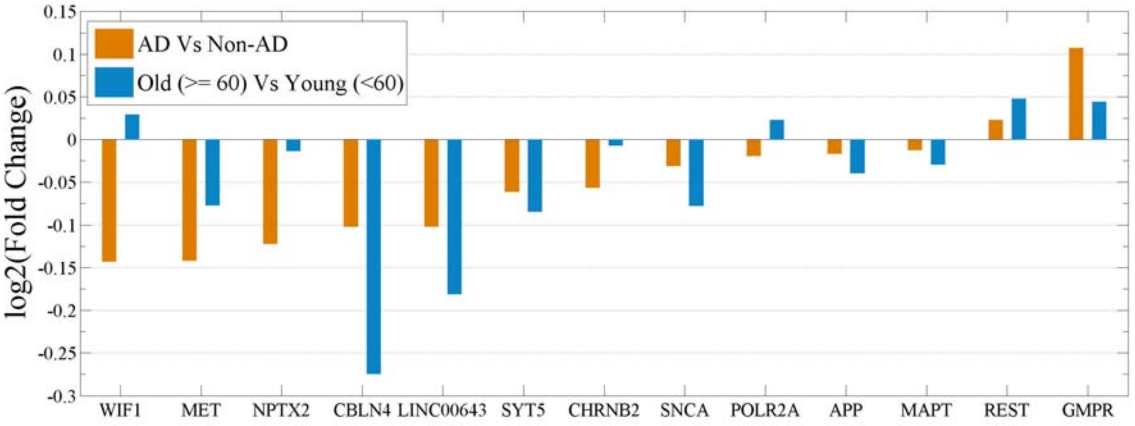
AD Vs Non-AD
Old (>= 60) Vs Young (<60)
log2(Fold Change)
0.15
0.1
0.05
0
-0.05
-0.1
-0.15
-0.2
-0.25
-0.3
WIF1
MET
NPTX2
CBLN4
LINC00643
SYT5
CHRNB2
SNCA
POLR2A
APP
MAPT
REST
GMPR

Figure S2

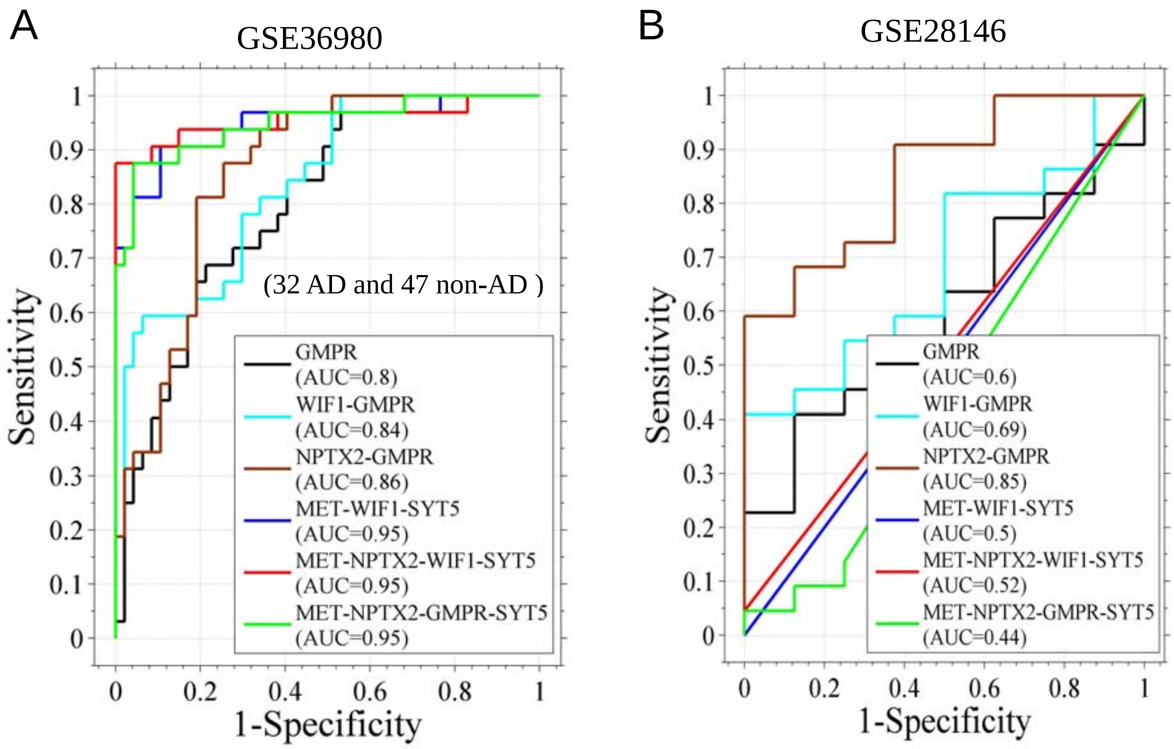
A
GSE36980
(32 AD and 47 non-AD)
Sensitivity
1-Specificity
GMPR
(AUC=0.8)
WIF1-GMPR
(AUC=0.84)
NPTX2-GMPR
(AUC=0.86)
MET-WIF1-SYT5
(AUC=0.95)
MET-NPTX2-WIF1-SYT5
(AUC=0.95)
MET-NPTX2-GMPR-SYT5
(AUC=0.95)
B
GSE28146
Sensitivity
1-Specificity
GMPR
(AUC=0.6)
WIF1-GMPR
(AUC=0.69)
NPTX2-GMPR
(AUC=0.85)
MET-WIF1-SYT5
(AUC=0.5)
MET-NPTX2-WIF1-SYT5
(AUC=0.52)
MET-NPTX2-GMPR-SYT5
(AUC=0.44)

Figure S3

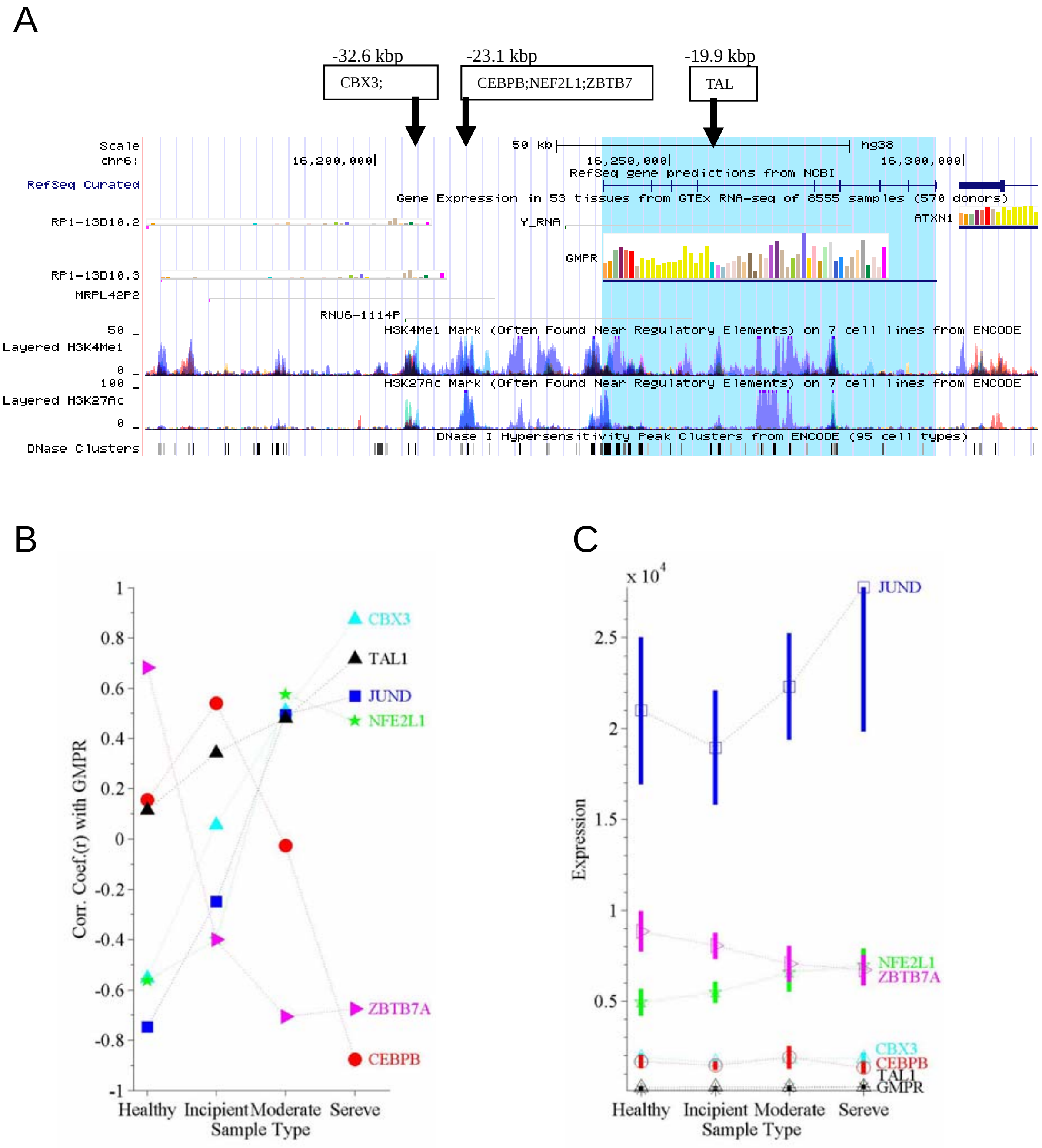

A
-32.6 kbp
CBX3;
-23.1 kbp
CEBPB;NEF2L1;ZBTB7
-19.9 kbp
TAL
Scale
chr6:
50 kb
hg38
16,200,000|
16,250,000|
16,300,000|
RefSeq gene predictions from NCBI
RefSeq Curated
Gene Expression in 53 tissues from GTEx RNA-seq of 8555 samples (570 donors)
RP1-13D10.2
Y_RNA
ATXN1
GMPR
RP1-13D10.3
MRPL42P2
RNU6-1114P
H3K4Me1 Mark (Often Found Near Regulatory Elements) on 7 cell lines from ENCODE
Layered H3K4Me1
H3K27Ac Mark (Often Found Near Regulatory Elements) on 7 cell lines from ENCODE
Layered H3K27Ac
DNase I Hypersensitivity Peak Clusters from ENCODE (95 cell types)
DNase Clusters
B
Corr. Coef.(r) with GMPR
CBX3
TAL1
JUND
NFE2L1
ZBTB7A
CEBPB
Healthy
Incipient
Moderate
Sereve
Sample Type
C
x 10^4
Expression
JUND
NFE2L1
ZBTB7A
CBX3
CEBPB
TAL1
GMPR
Healthy
Incipient
Moderate
Sereve
Sample Type

Figure S4

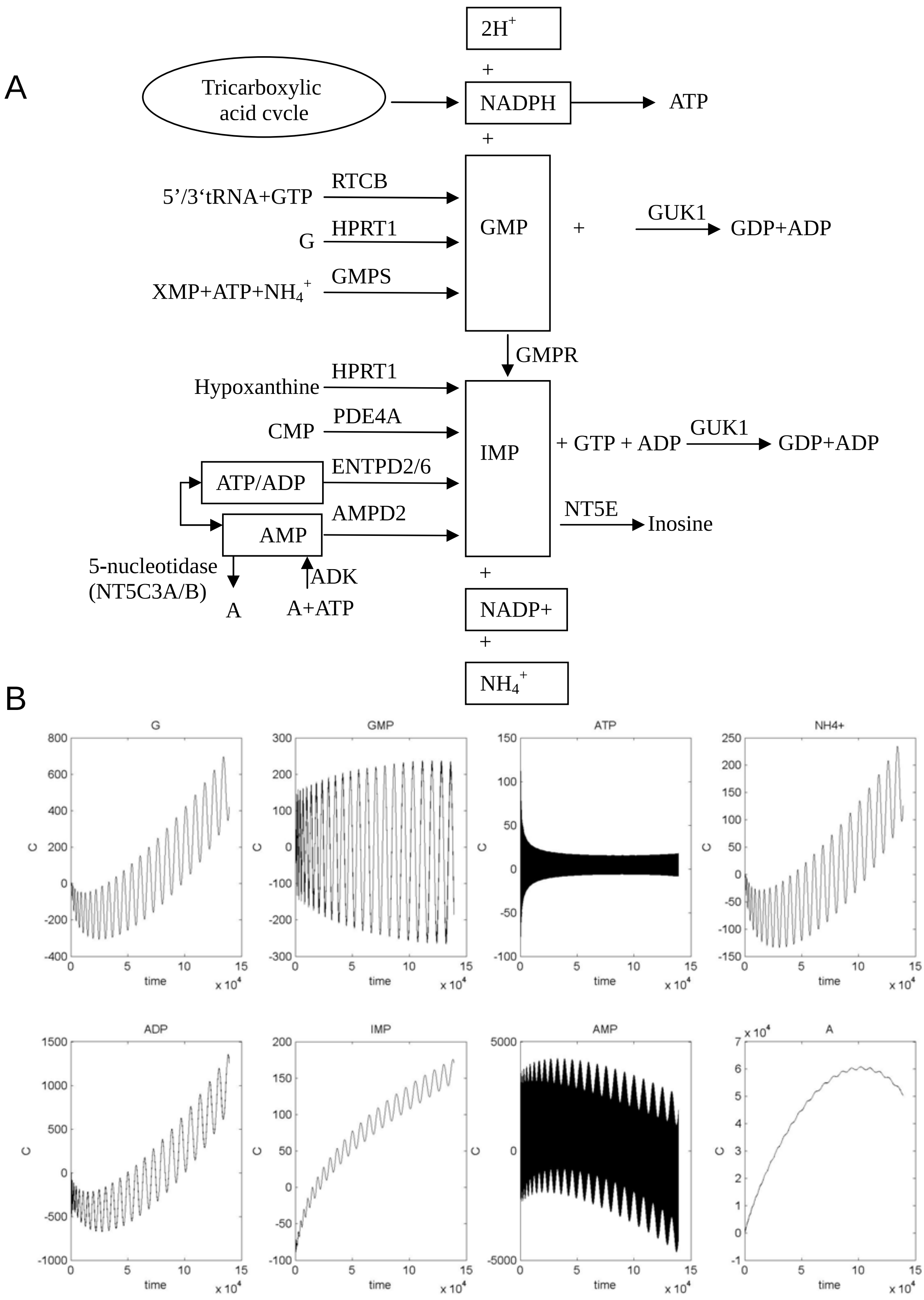
A
2H+
+
Tricarboxylic
acid cvcle
NADPH
ATP
+
RTCB
5'/3'tRNA+GTP
HPRT1
G
GMPS
XMP+ATP+NH4+
GMP
+
GUK1
GDP+ADP
GMPR
HPRT1
Hypoxanthine
PDE4A
CMP
ENTPD2/6
ATP/ADP
AMPD2
AMP
IMP
+ GTP + ADP
GUK1
GDP+ADP
NT5E
Inosine
5-nucleotidase
(NT5C3A/B)
A
ADK
A+ATP
+
NADP+
+
NH4+
B
G
GMP
ATP
NH4+
ADP
IMP
AMP
A
C
time
x 10^4

Figure S5

A

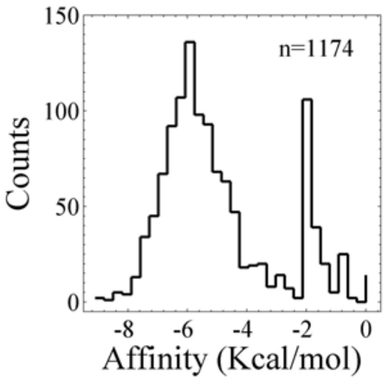

B

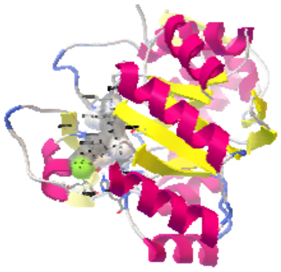

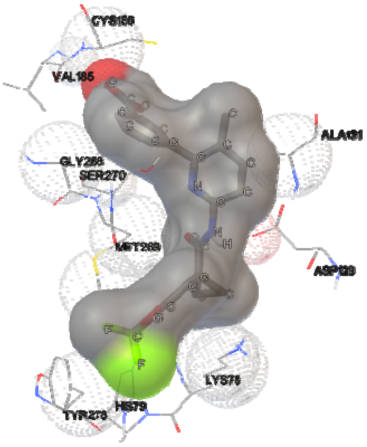

GMPR2-Lumacaftor
ΔG=−10.4Kcal/mol
P-value=0.0163
H-Bond:VAL185…HN

Figure S6

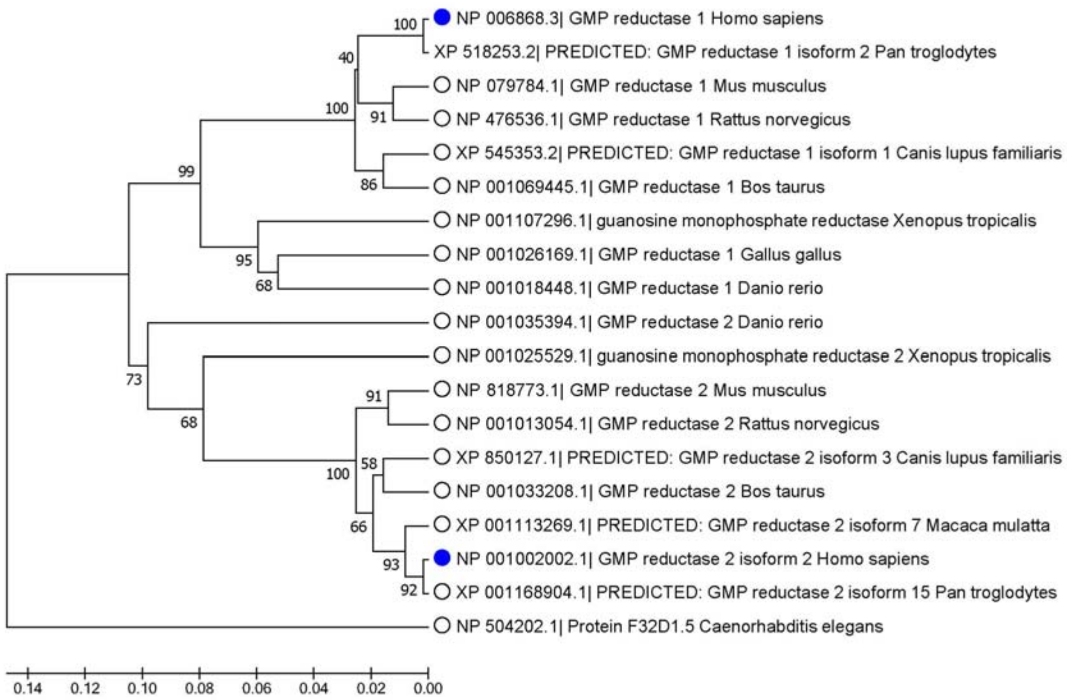
NP 006868.3| GMP reductase 1 Homo sapiens
XP 518253.2| PREDICTED: GMP reductase 1 isoform 2 Pan troglodytes
NP 079784.1| GMP reductase 1 Mus musculus
NP 476536.1| GMP reductase 1 Rattus norvegicus
XP 545353.2| PREDICTED: GMP reductase 1 isoform 1 Canis lupus familiaris
NP 001069445.1| GMP reductase 1 Bos taurus
NP 001107296.1| guanosine monophosphate reductase Xenopus tropicalis
NP 001026169.1| GMP reductase 1 Gallus gallus
NP 001018448.1| GMP reductase 1 Danio rerio
NP 001035394.1| GMP reductase 2 Danio rerio
NP 001025529.1| guanosine monophosphate reductase 2 Xenopus tropicalis
NP 818773.1| GMP reductase 2 Mus musculus
NP 001013054.1| GMP reductase 2 Rattus norvegicus
XP 850127.1| PREDICTED: GMP reductase 2 isoform 3 Canis lupus familiaris
NP 001033208.1| GMP reductase 2 Bos taurus
XP 001113269.1| PREDICTED: GMP reductase 2 isoform 7 Macaca mulatta
NP 001002002.1| GMP reductase 2 isoform 2 Homo sapiens
XP 001168904.1| PREDICTED: GMP reductase 2 isoform 15 Pan troglodytes
NP 504202.1| Protein F32D1.5 Caenorhabditis elegans
100
40
100
91
99
86
95
68
73
68
91
100
58
66
93
92
0.14
0.12
0.10
0.08
0.06
0.04
0.02
0.00

Figure S7

GMPR1 GMPR2

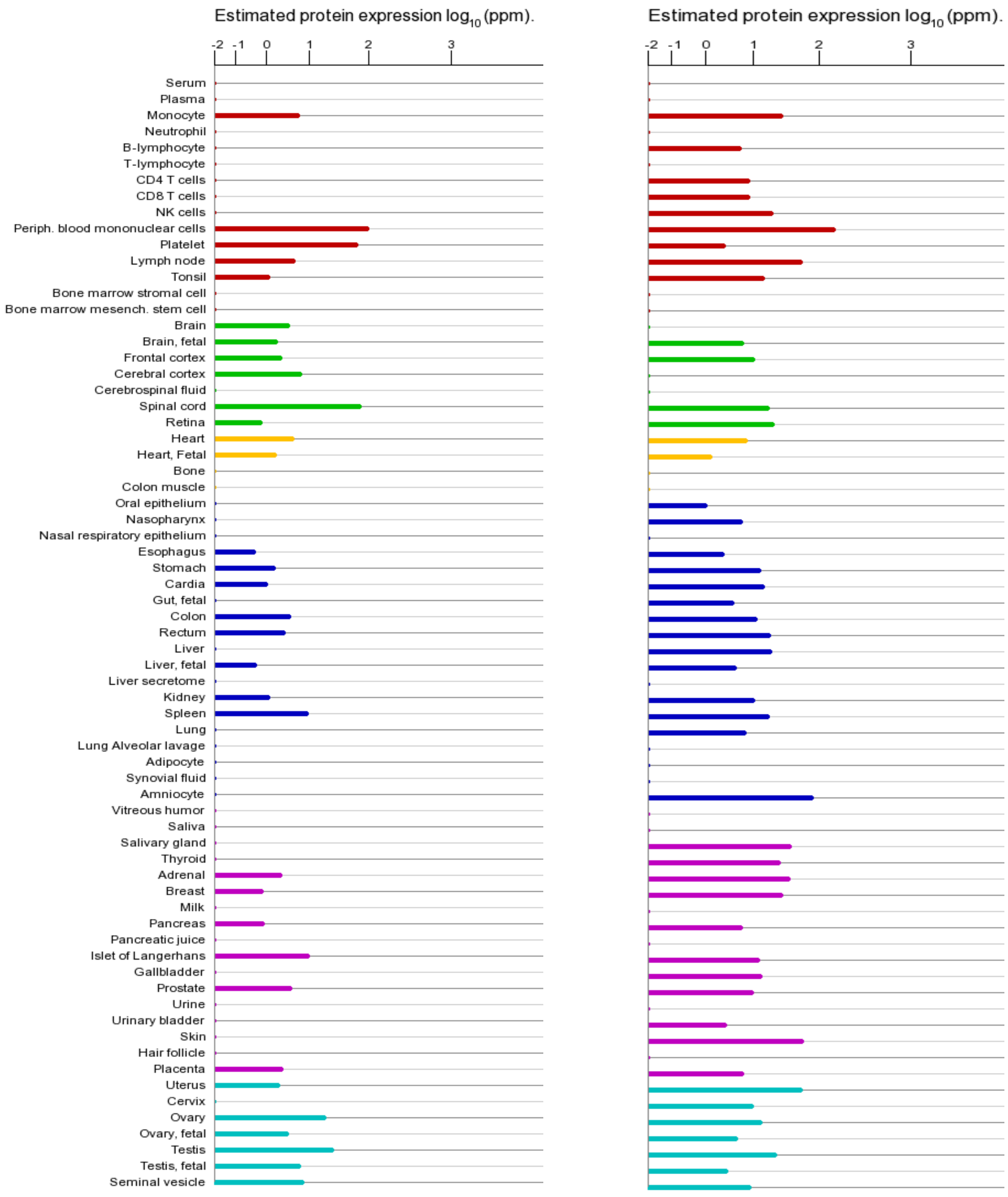

Supplement: Supplementary file 1 — Supplementary Information [file 41598_2018_21256_MOESM1_ESM.pdf]
